# Scatter Hoarding of Seeds Confers Survival Advantages and Disadvantages to Large-Seeded Tropical Plants at Different Life Stages

**DOI:** 10.1371/journal.pone.0124932

**Published:** 2015-05-13

**Authors:** Erin K. Kuprewicz

**Affiliations:** Department of Biology, University of Miami, Coral Gables, Florida, United States of America; DOE Pacific Northwest National Laboratory, UNITED STATES

## Abstract

Scatter hoarding of seeds by animals contributes significantly to forest-level processes, including plant recruitment and forest community composition. However, the potential positive and negative effects of caching on seed survival, germination success, and seedling survival have rarely been assessed through experimental studies. Here, I tested the hypothesis that seed burial mimicking caches made by scatter hoarding Central American agoutis (*Dasyprocta punctate*) enhances seed survival, germination, and growth by protecting seeds from seed predators and providing favorable microhabitats for germination. In a series of experiments, I used simulated agouti seed caches to assess how hoarding affects seed predation by ground-dwelling invertebrates and vertebrates for four plant species. I tracked germination and seedling growth of intact and beetle-infested seeds and, using exclosures, monitored the effects of mammals on seedling survival through time. All experiments were conducted over three years in a lowland wet forest in Costa Rica. The majority of hoarded palm seeds escaped predation by both invertebrates and vertebrates while exposed seeds suffered high levels of infestation and removal. Hoarding had no effect on infestation rates of *D*. *panamensis*, but burial negatively affected germination success by preventing endocarp dehiscence. Non-infested palm seeds had higher germination success and produced larger seedlings than infested seeds. Seedlings of *A*. *alatum* and *I*. *deltoidea* suffered high mortality by seed-eating mammals. Hoarding protected most seeds from predators and enhanced germination success (except for *D*. *panamensis*) and seedling growth, although mammals killed many seedlings of two plant species; all seedling deaths were due to seed removal from the plant base. Using experimental caches, this study shows that scatter hoarding is beneficial to most seeds and may positively affect plant propagation in tropical forests, although tradeoffs in seed survival do exist.

## Introduction

Seed predation by animals negatively affects plant populations by limiting plant propagation and also may influence forest community structure and plant distributions [1–4]. Once a seed lands on the forest floor, it is susceptible to attack by a suite of terrestrial seed-eating animals including invertebrates and vertebrates [5]. In neotropical rain forests, many beetles in the family Bruchidae and subfamily Scolytinae attack and kill large seeds, contributing to high levels of seed mortality [5–8]. Neotropical rodents (e.g., agoutis, squirrels, rats) and ungulates (e.g., peccaries, tapirs) also consume, and may potentially disperse, a variety of large seeds [9–11].

Although seed predators negatively affect plant recruitment via seed consumption and destruction, mammals can positively affect seed survival and propagation by dispersing seeds away from source plants (e.g., [12–14]). Dispersal away from the parent plant may increase the likelihood of a seed escaping predation [15–18] and may enhance germination success if a seed is deposited in a favorable microhabitat and subsequently abandoned by the disperser [17, 19, 20].

Some animals disperse seeds by hoarding them in subsurface caches, conserving them for future use [14, 21]. Consumption of a hoarded seed is temporally deferred and the cached item is transported, deposited, and concealed in a way that prevents detection by kleptoparasitic seed predators [22]. Scatter hoarders store single food items within numerous caches located throughout their home ranges [23]. Previous research on scatter hoarding by mammals has focused only on the positive effects that hoarding has on seeds and has been mostly observational in nature (e.g., [12, 19, 22, 24]); the potential damaging effects of hoarding have been poorly documented. Experiments explicitly testing if and how hoarding affects the survival of seeds and recruitment of seedlings are especially sparse. Hoarded seeds are usually dispersed long distances away from parent trees, enhancing the probability that these seeds will escape detection by other granivores [12, 21, 22]. Scattered, shallow caches hide plant propagules from seed predators and may provide favorable microsites for germination and seedling establishment [21]. Scatter hoarding, however, also may negatively affect seeds if they are hoarded in unsuitable habitats or buried too deep within the soil, preventing germination and seedling emergence [21].

Central American agoutis (*Dasyprocta punctata* Gray, 1842; Rodentia) are large (2–4 kg) terrestrial mammals that consume, disperse, and scatter hoard seeds of numerous large-seeded plants [22, 25]. Agouti scatter-hoarding behavior has been well-documented [22, 23, 25], but few studies have experimentally quantified the suite of effects (positive or negative) that this hoarding activity has on seed survival, germination, and resultant seedling growth and survival.

Plant mortality is usually highest during seed and young seedling stages [3, 26]. In addition to the seed stage, seedlings are susceptible to damage and destruction by large terrestrial mammals in the understory via trampling [27], herbivory [28], or seed predation [12, 29, 30]. Understory ungulate species that forage in large groups (e.g., collared peccaries, *Pecari tajacu*) can trample or incidentally bury seedlings while searching for food on the forest floor. Moreover, mammal herbivores also directly consume young seedlings [9, 28]. Small seedlings may act as indicators of below ground seed presence and, using this cue, mammals excavate and remove the attached seed resulting in incidental seedling death [12, 29, 30].

In this study I tested the hypothesis that scatter hoarding positively affects seeds by protecting them from seed predators and by enhancing interred seed germination and resultant seedling growth. However, once seeds germinate and young seedlings emerge on the forest floor, they are no longer hidden from seed predators, and seedlings and their attached seeds are susceptible to attack by large generalist mammal herbivore-granivores. To test this hypothesis, the main objectives of my research were: (1) to determine if hoarding effectively protects plant propagules from detection and subsequent destruction by invertebrate and vertebrate seed predators, (2) to compare the germination success and seedling growth of palm seeds (*Astrocaryum alatum* H. F. Loomis, *Iriartea deltoidea* Ruiz & Pav., *Socratea exorrhiza* (Mart.) H. Wendl.) infested (non-hoarded) versus those non-infested (hoarded) by invertebrates, (3) to ascertain if hoarding by agoutis positively or negatively affects the germination of *Dipteryx panamensis* (Pittier) Record & Mell seeds, and (4) to determine how large terrestrial mammals affect the survival of young, recently-germinated seedlings.

## Materials and Methods

### Ethics Statement

This research was conducted in accordance with the laws of the Costa Rican government (MINAE-SINAC, Ministerio del Ambiente y Energía-Sistema Nacional de Áreas de Conservación) to work with wild animals and plants in the Área de Conservación Cordillera Volcánica Central (Resolución N° 072-2006-SINAC, 153-2007-SINAC, 135-2007-SINAC, 086-2008-SINAC). All work with animals was observational rather than invasive and complied with all ethical and legal requirements of the Institutional Animal Care and Use Committee at the University of Miami (ACUC Protocol Number 06–013). All work was conducted within La Selva Biological Station under the approval of the Station Director, Dr. Deedra McClearn and the Organization for Tropical Studies.

### Study site and species

This study was conducted from November 2006 to March 2009 at La Selva Biological Station (hereafter La Selva) located in Puerto Viejo, Sarapiquí, Heredia, Costa Rica (10°26' N, 83°59' W). La Selva is a protected reserve classified as tropical lowland wet forest [31], which comprises 1600 ha of old-growth forest, secondary growth, swamps, and tree plantations. This area receives approximately 4000 mm of rainfall per year and is aseasonal, having no distinct dry season [32]. I conducted this study throughout the primary forest of La Selva, which corresponds to the habitat where the focal plant species are most abundant and where agoutis and peccaries are the most common terrestrial mammal frugivores (TEAM Network, http://www.teamnetwork.org/en/) [33].

For hoarding experiments, I used fresh, ripe seeds of *A*. *alatum*, *I*. *deltoidea*, *S*. *exorrhiza* (all Arecaceae), and entire fruits of *D*. *panamensis* (Fabaceae) (Table 1). Agoutis cache *D*. *panamensis* seeds while they are still enclosed within endocarps [34], (E. Kuprewicz, pers. obs.). Seeds of these tree species were chosen for use in experiments because of their large sizes and abundance throughout the primary forest of La Selva; these species are also readily eaten by agoutis, peccaries, squirrels, and small rodents and are scatter hoarded by agoutis [11]. Agoutis and peccaries do not pass any of these large seeds through their guts intact (endozoochory), rather they pulverize seeds prior to ingesting them, resulting in embryo destruction and seed death.

**Table 1:** Seed and fruit characteristics of all plant species used in hoarding experiments.

Species	Diaspore type	Family	Fruiting period	Mass (g) Mean ± 1 SD	Diaspore dimensions
*Astrocaryum alatum*	Seed	Arecaceae	year-round	25.1 ± 4.5	length = 6 cm, width = 4 cm
*Iriartea deltoidea*	Seed	Arecaceae	year-round	2.9 ± 0.9	diameter = 2–2.8 cm
*Socratea exorrhiza*	Seed	Arecaceae	year-round; peak Oct.–Dec.	3.6 ± 0.6	length = 2.5–3.5 cm, diameter = 1.5–2 cm
*Dipteryx panamensis*	Fruit	Fabaceae	Nov.–March	25.2 ± 4.4	length = 6 cm, width = 3 cm

Sample sizes: *Astrocaryum alatum n* = 104, *Iriartea deltoidea n* = 50, *Socratea exorrhiza n* = 69, *Dipteryx panamensis n* = 15

All data considered in this study (and associated documentation for use) are archived in the open-access figshare repository at http://dx.doi.org/10.6084/m9.figshare.1256359

### Experiment 1: The effects of hoarding on seed detection by invertebrate and vertebrate seed predators

For hoarding experiments, I collected ripe fallen seeds and fruits during the peak fruiting seasons of each species. I collected fruits from at least 15 individual trees per species. Seeds were thereafter pooled by species and a subset was randomly chosen for use in hoarding experiments. Hoarding experiments were completed during the months when each plant species was fruiting, so as to coincide with the time period when ambient abundances of their respective fruits were highest (Table 1).

For all experiments, I used seeds of similar sizes and masses (with respect to species) with no evidence of insect infestation or fungus growth. To detect insect infestation, I visually inspected each seed for small holes (indicative of infestation by scolytine beetles) or large holes (indicating infestation by bruchid beetles); any seeds with apparent holes were discarded.

**Fig 1:**
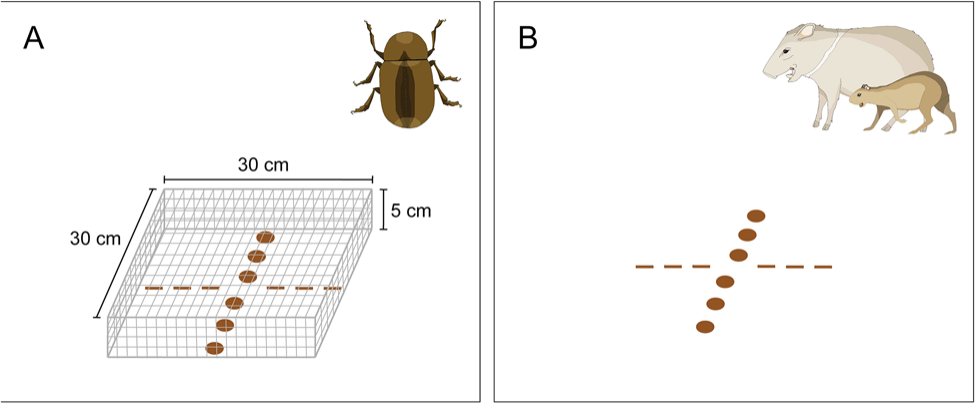
Schematic illustration of hoarding depots used to assess infestation by insects and removal by mammals for hoarded and non-hoarded seeds. The invertebrate treatment (A) is characterized by an exclosure that prevents mammal access to seeds yet allows invertebrate access; the vertebrate treatment (B) has no exclosure, allowing mammals to access and remove seeds. Six seeds were placed on the soil surface (ovals) and six seeds were buried 5 cm below the soil to mimic agouti seed caches (dashes). Each depot (either a single invertebrate or vertebrate treatment) was placed within primary forest at least 300 m from other depots. All illustrations by E. K. Kuprewicz.

Prior to placement in the field, I removed all pulp from each seed of *A*. *alatum*, *I*. *deltoidea*, and *S*. *exorrhiza* to mimic agouti hoarding behavior for these species (E. Kuprewicz, pers. obs.). However, for *D*. *panamensis*, agoutis hoard the entire fruit pod (exocarp, endocarp, and seed), therefore I left these fruits intact for experiments. Twelve seeds of a single species were placed in each hoarding depot; six seeds were buried 5 cm beneath the soil (mean depth of agouti seed hoards, [25]) and six seeds remained exposed on the soil surface (Fig 1). Each seed was separated from neighboring seeds by 4 cm. I lightly pressed the soil down over hoarded seeds to mimic the soil compacting behavior of agoutis [25], (E. Kuprewicz, pers. obs.). I covered seeds and fruits at a portion (45%) of the total number of hoarding depots with a wire mesh cage (length = 30 cm, width = 30 cm, height = 5 cm, mesh opening size = 2 × 2 cm) staked firmly into the soil to prevent seed removal by mammals but allowing insect access to the seeds (invertebrate treatment, Fig 1A). Identical seed arrangements without cages were open to seed-eating mammals (vertebrate treatment, Fig 1B). Hoarding depots (either invertebrate or vertebrate treatments) were placed singly and separated by a minimum distance of 300 m to prevent agouti home range overlap with more than one depot (agouti home range estimates are typically smaller than 300 m^2^; [25]). I placed the following numbers of hoarding depots (caged, invertebrate access treatment / non-caged, vertebrate access treatment) throughout the primary forest of La Selva for each species: *A*. *alatum*: 11 / 10; *I*. *deltoidea*: 10 / 10; *S*. *exorrhiza*: 10 / 18; and *D*. *panamensis*: 10 / 10.

All hoarding depots (both invertebrate and vertebrate treatments) remained in the field for 36 d periods (seeds on the forest floor are encountered by terrestrial mammals and insects within this time, [11]). Thereafter, I assessed (1) seed and fruit infestation by insects in the caged, invertebrate access treatment and (2) removal by mammals in the non-caged, vertebrate access treatment. I compared infestation by insects (caged treatment) and removal by mammals (non-caged treatment) for all hoarded and non-hoarded seeds and fruits using paired t-tests. All statistical analyses were performed in R (version 3.1.0, 2014). I collected all intact seeds and fruits from the field for germination and growth experiments.

### Experiment 2a: Germination success and seedling growth of palm seeds infested by invertebrates

I planted infested and non-infested seeds of *A*. *alatum*, *I*. *deltoidea*, and *S*. *exorrhiza* in individual germination bags filled with soil gathered from one location within the primary forest to ensure a consistent growth medium for all seeds. Invertebrates did not infest fruits of *D*. *panamensis* so this species was excluded from this portion of the study. Palm seeds were exposed to homogeneous natural light, rain, and soil conditions within a shade house (76% shade). I recorded germination success (production of radicle and plumule) of infested and non-infested seeds for all palm species. I compared germination success of infested and non-infested palm seeds using Fisher exact tests (for 2 × 2 matrices of count data for each species). I also measured seedling height (= length of longest leaf) after a growth period of 120 d and I compared the heights of infested and non-infested *S*. *exorrhiza* seedlings (the only palm species in which infested seeds germinated) using Welch’s two sample t-test.

### Experiment 2b: Germination success of hoarded and non-hoarded *D*. *panamensis* seeds

*Dipteryx panamensis* seeds germinate rapidly relative to the aforementioned palm seeds, therefore I assessed how experimental burial (mimicking hoarding by agoutis) affects germination success, rather than seedling growth. After exposure to animals in the field for 36 d, I brought all hoarded and non-hoarded *D*. *panamensis* fruits to a shade house to prevent further animal interference. Hoarded fruits were carefully excavated with their surrounding soil and immediately transferred to the shade house while still in a buried state. Depending on their previous state in the field (hoarded or non-hoarded), fruits were either reburied 5 cm below the soil (hoarded) or placed on the soil surface (non-hoarded) in soil-filled germination trays for an additional 14 d to allow adequate time for seedling germination (no seeds had germinated within 36 d). I recorded germination for each seed after 50 d and compared the germination success between treatments (hoarded versus non-hoarded) using Fisher exact tests (for 2 × 2 matrices of count data).

### Experiment 3: The effects of large terrestrial mammals on young seedling survival

For seedling experiments, I grew seedlings from freshly harvested seeds of *A*. *alatum*, *I*. *deltoidea*, *S*. *exorrhiza*, and *D*. *panamensis*. To determine how large terrestrial mammals affect the survival of these large-seeded tree seedlings, I planted seedling pairs of each plant species throughout the primary forest of La Selva (*A*. *alatum n* = 18 pairs, *I*. *deltoidea n* = 17 pairs, *S*. *exorrhiza n* = 19 pairs, *D*. *panamensis n* = 20 pairs). Seedlings of all species were age and size-matched for placement in the field. *Astrocaryum alatum*, *I*. *deltoidea*, and *S*. *exorrhiza* seedlings retained nutrient sources (seeds), whereas *D*. *panamensis* seedlings retained cotyledons when they were planted in the forest.

To determine how large terrestrial mammals affect seedling survival, I placed *A*. *alatum*, *I*. *deltoidea*, *S*. *exorrhiza*, and *D*. *panamensis* seedlings within and outside mammal exclosures and monitored their survival. One seedling of each pair was enclosed within a cylindrical mammal exclosure made of metal mesh (diameter = 25 cm; height = 1 m; mesh opening size = 2 × 2 cm); the other seedling was planted 1 m from the caged seedling and remained open and exposed (non-caged) to all seedling predators. Mammal exclosures prevented access by all terrestrial mammals, but allowed access to seedlings by invertebrates, fungal spores, and other pathogens. Paired seedlings were separated from other pairs by at least 300 m to ensure sample independence.

I checked seedling pairs every 14 d and noted survival status (alive or dead) of each caged and non-caged seedling for a total of 140 d. I compared the numbers of surviving caged and non-caged seedlings at the end of the experiment using chi-squared tests.

## Results

### Experiment 1: The effects of hoarding on seed detection by invertebrate and vertebrate seed predators

**Fig 2:**
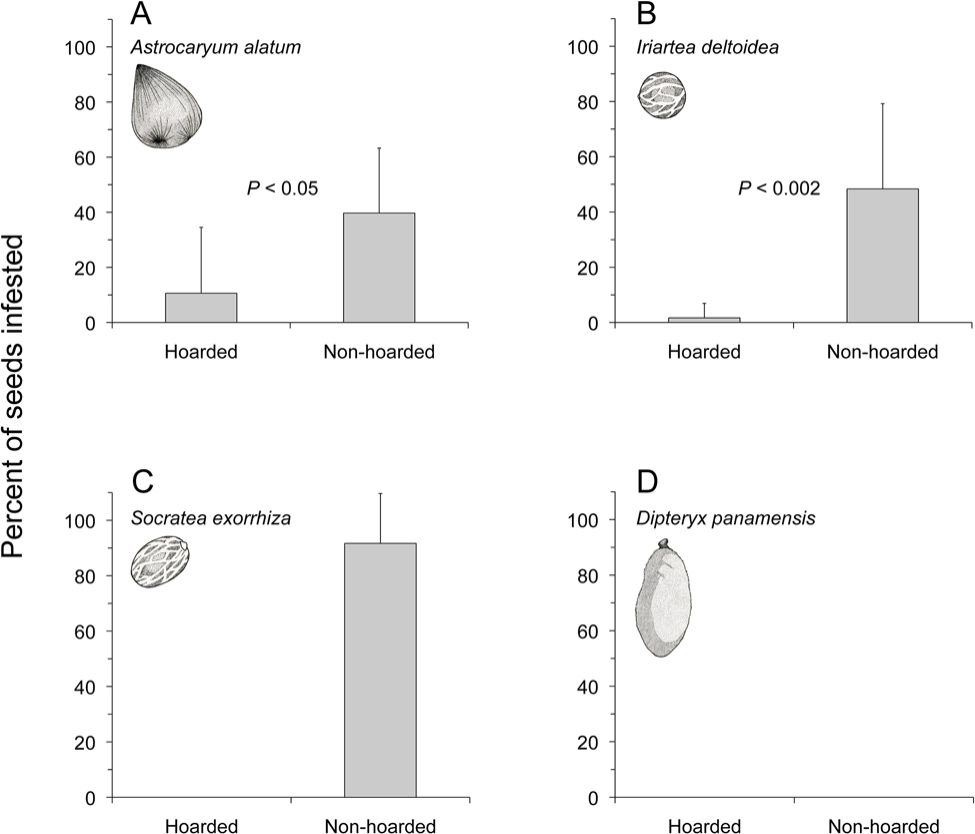
Invertebrate infestation of seeds after either 36 d of burial under 5 cm of soil (hoarded) or exposure on the soil surface (non-hoarded) for seeds of *A*. *alatum* (A), *I*. *deltoidea* (B), *S*. *exorrhiza* (C), and fruits of *D*. *panamensis* (D). *n* = 11 depots for *A*. *alatum* and *n* = 10 depots for *I*. *deltoidea*, *S*. *exorrhiza*, and *D*. *panamensis*. Experimental setup as in Fig 1A. Bars represent mean percent of seeds infested in each depot + 1 SD.

After 36 d of exposure on the primary forest floor, 59% of seeds from the three palm species were infested by beetles of the genus *Coccotrypes* (Coleoptera: Curculionidae: Scolytinae). These small (approximately 1 mm in length) beetles bored holes through the seed coat and into the endosperm of the palm seeds. Hoarded seeds of all three palm species were protected from infestation by invertebrate seed predators whereas non-hoarded seeds suffered significantly higher levels of infestation by *Coccotrypes* beetles (*A*. *alatum*: *t* = -2.25, df = 10, *P* < 0.048; *I*. *deltoidea*: *t* = -4.45, df = 9, *P* < 0.002; *S*. *exorrhiza*: no statistical test needed for obvious difference between treatments, *n* = 10 depots; Fig 2A, 2B and 2C). There was no evidence of infestation by *Coccotrypes* beetles or other seed-boring insects in any of the hoarded or non-hoarded *D*. *panamensis* fruits (Fig 2D).

**Fig 3:**
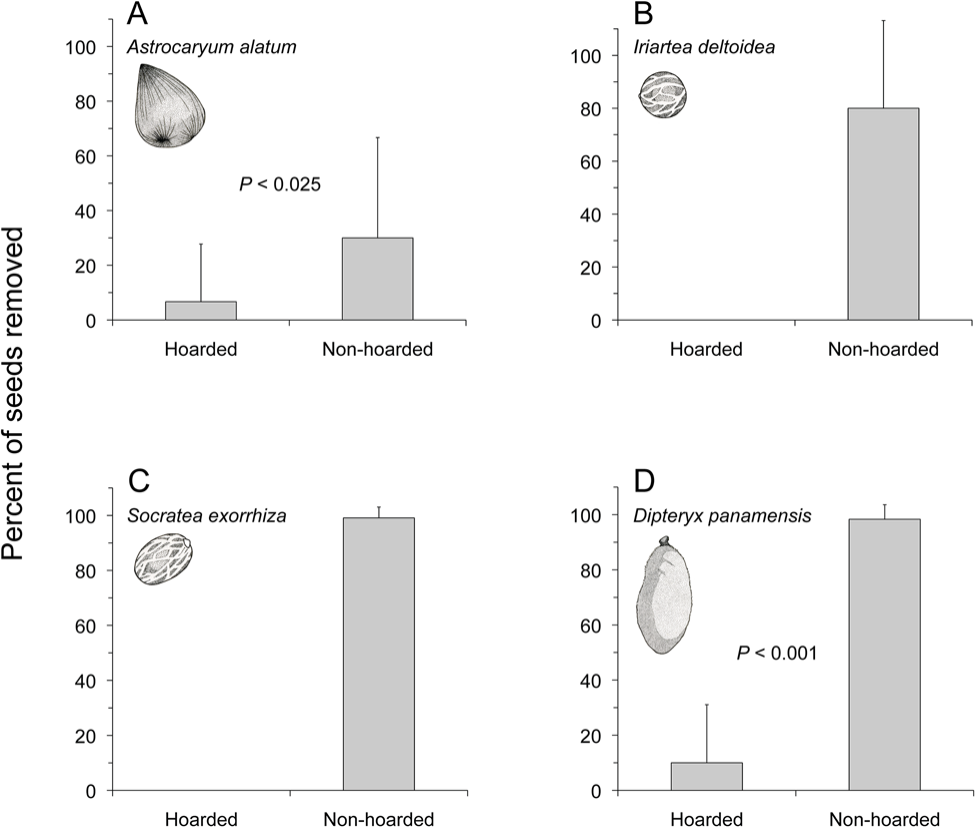
Removal by terrestrial mammals of seeds after either 36 d of burial under 5 cm of soil (hoarded) or exposure on the soil surface (non-hoarded) for seeds of *A*. *alatum* (A), *I*. *deltoidea* (B), *S*. *exorrhiza* (C), and fruits of *D*. *panamensis* (D). *n* = 10 depots for *A*. *alatum*, *I*. *deltoidea*, and *D*. *panamensis*, and *n* = 18 depots for *S*. *exorrhiza*. Experimental setup as in Fig 1B. Bars represent mean percent of seeds removed from each depot + 1 SD.

The large sizes of all propagules used in this study precluded their removal by small mammals (e.g., *Heteromys desmarestianus*, *Proechimys semispinosus*); most mammal interaction with seeds was by peccaries and agoutis (confirmed by field observations and camera traps deployed at a subset of seed depots). After 36 d, these large mammals had visited all hoarding depots (vertebrate treatment) and removed seeds and fruits of all four plant species. Overall, hoarded food items (seeds and fruits) had significantly lower levels of removal by mammals than non-hoarded food items (*A*. *alatum*: *t* = -2.69, df = 9, *P* < 0.025; *I*. *deltoidea*: no statistical test needed for obvious difference between treatments, *n* = 10 depots; *S*. *exorrhiza*: no statistical test needed for obvious difference between treatments, *n* = 18 depots; *D*. *panamensis*: *t* = -12.53, df = 9, *P* < 0.001; Fig 3).

### Experiment 2a: Germination success and seedling growth of palm seeds infested by invertebrates

**Fig 4:**
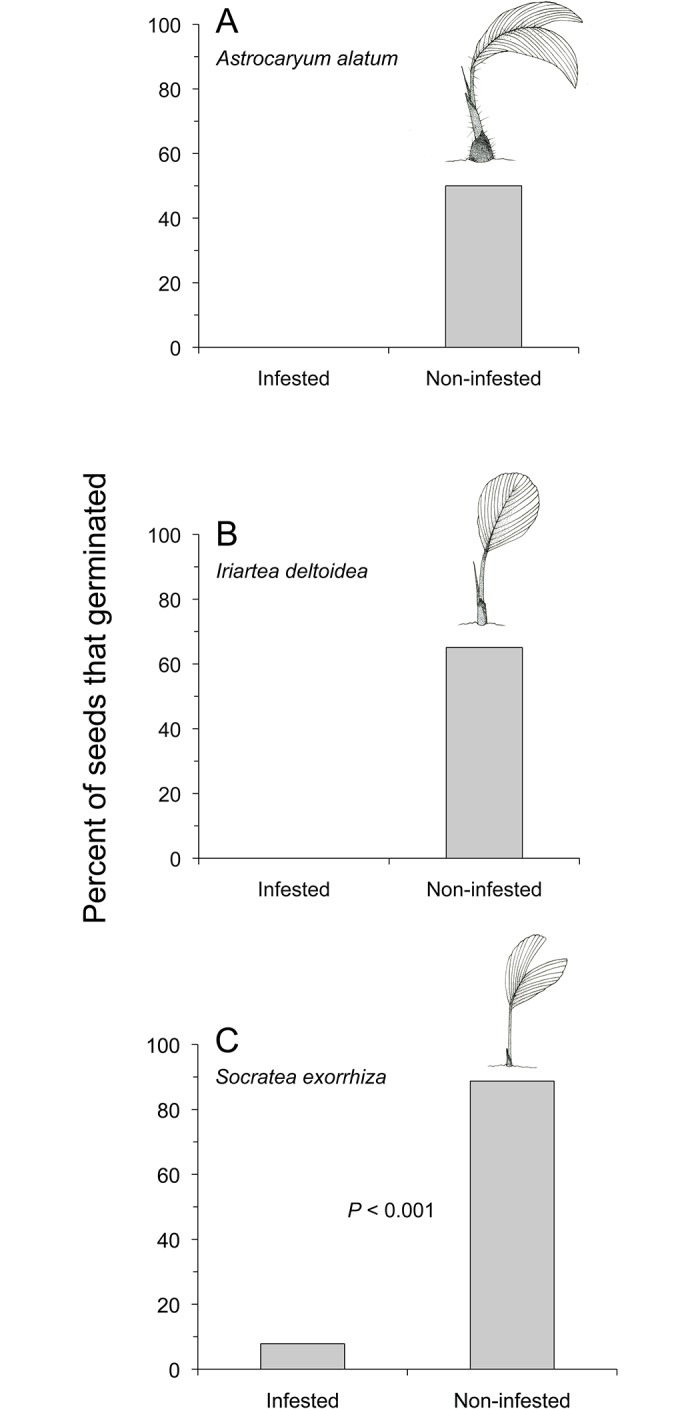
Germination success of palm seeds either infested or non-infested by beetles (*Coccotrypes* spp.) after 120 d. Sample sizes: *A*. *alatum* seeds (A, *n*_infested, germinated_ = 0, *n*_non-infested, germinated_ = 16, *n*_infested, non-germinated_ = 4, *n*_non-infested, non-germinated_ = 16), *I*. *deltoidea* seeds (B, *n*_infested, germinated_ = 0, *n*_non-infested, germinated_ = 41, *n*_infested, non-germinated_ = 59, *n*_non-infested, non-germinated_ = 22), and *S*. *exorrhiza* seeds (C, *n*_infested, germinated_ = 4, *n*_non-infested, germinated_ = 63, *n*_infested, non-germinated_ = 47, *n*_non-infested, non-germinated_ = 8).

Infested seeds of *A*. *alatum* and *I*. *deltoidea* did not germinate and infested seeds of *S*. *exorrhiza* had lower germination success than seeds that had not been attacked by *Coccotrypes* (*A*. *alatum*: Fig 4A; *I*. *deltoidea*: Fig 4B; *S*. *exorrhiza*: Fisher exact test, *P* < 0.001, Fig 4C).

**Fig 5:**
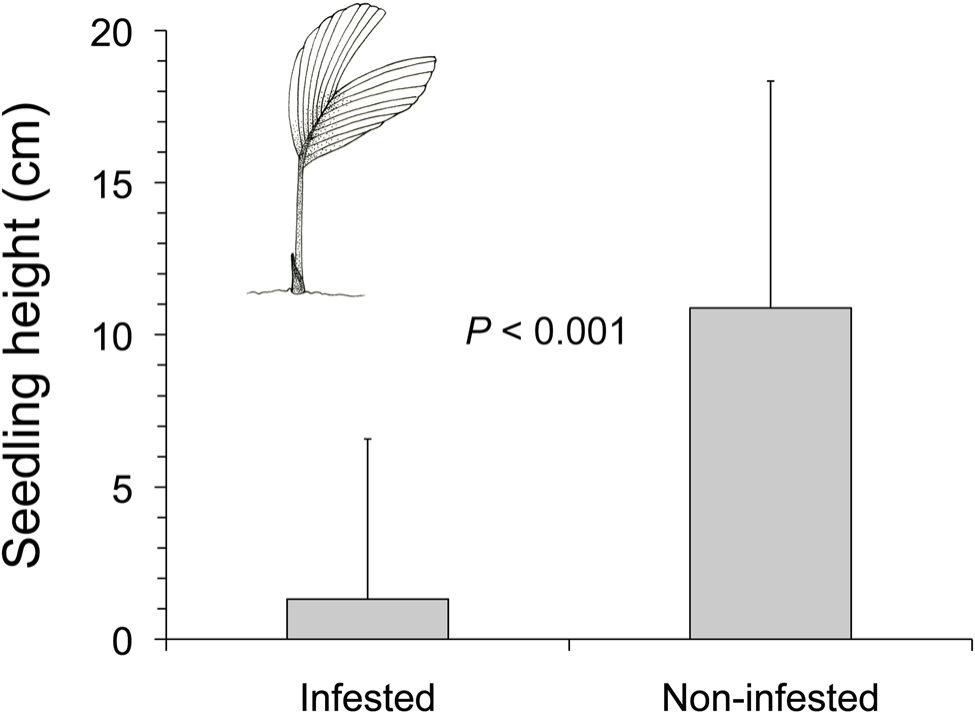
Heights of *S*. *exorrhiza* seedlings produced from seeds infested by *Coccotrypes* beetles or non-infested seeds. Seedlings were measured after 120 d of growth in a shade house under natural light and rainfall conditions. *n*_infested_ = 51 seedlings, *n*_non-infested_ = 71 seedlings. Please note that none of the infested seeds of *A*. *alatum* or *I*. *deltoidea* were able to germinate. Bars represent mean heights of seedlings + 1 SD.

Seedlings produced from *S*. *exorrhiza* seeds infested by *Coccotrypes* beetles were shorter than seedlings produced from non-infested seeds (Welch’s two sample t-test, *t* = -8.29, df = 120, *P* < 0.001; Fig 5).

### Experiment 2b: Germination success of hoarded and non-hoarded *D*. *panamensis* seeds

**Fig 6:**
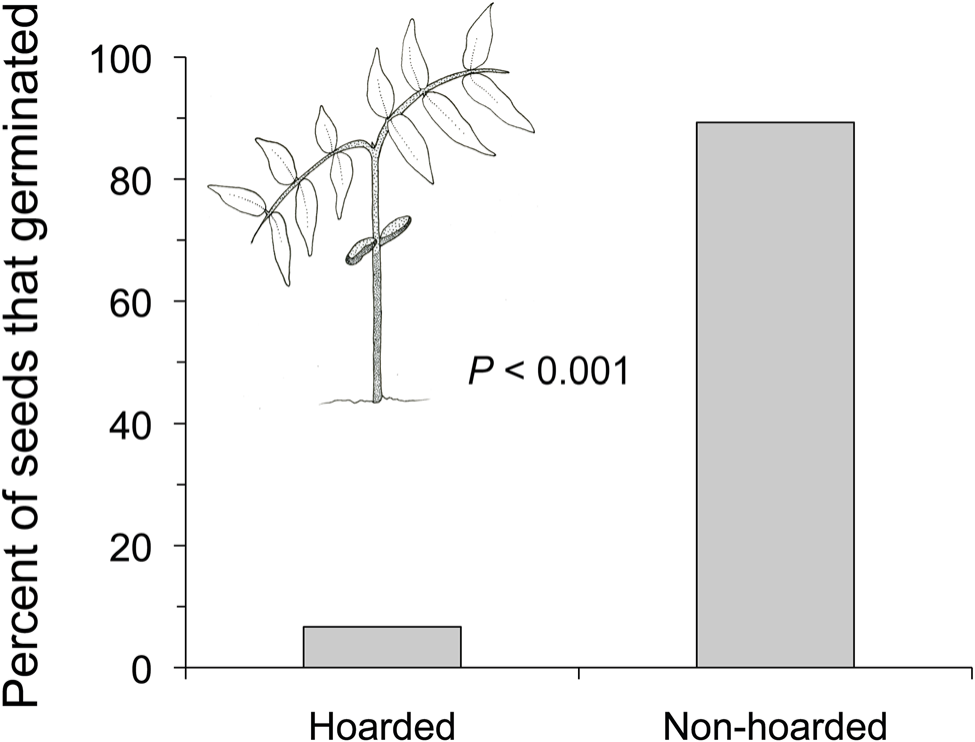
Germination success of hoarded and non-hoarded *D*. *panamensis* fruits after either 50 d of burial under 5 cm of soil (hoarded) or exposure on the soil surface (non-hoarded). *n*_hoarded, germinated_ = 2, *n*_non-hoarded, germinated_ = 25, *n*_hoarded, non-germinated_ = 28, *n*_non-hoarded, non-germinated_ = 3.

Burial had a detrimental effect on the germination success of *D*. *panamensis* seeds, resulting in fewer seeds germinating from hoarded fruits than from non-hoarded fruits after 50 d (Fisher exact test, *n*_hoarded_ = 30 fruits, *n*_non-hoarded_ = 28 fruits) *P* < 0.001; Fig 6).

### Experiment 3: The effects of large terrestrial mammals on young seedling survival

**Fig 7:**
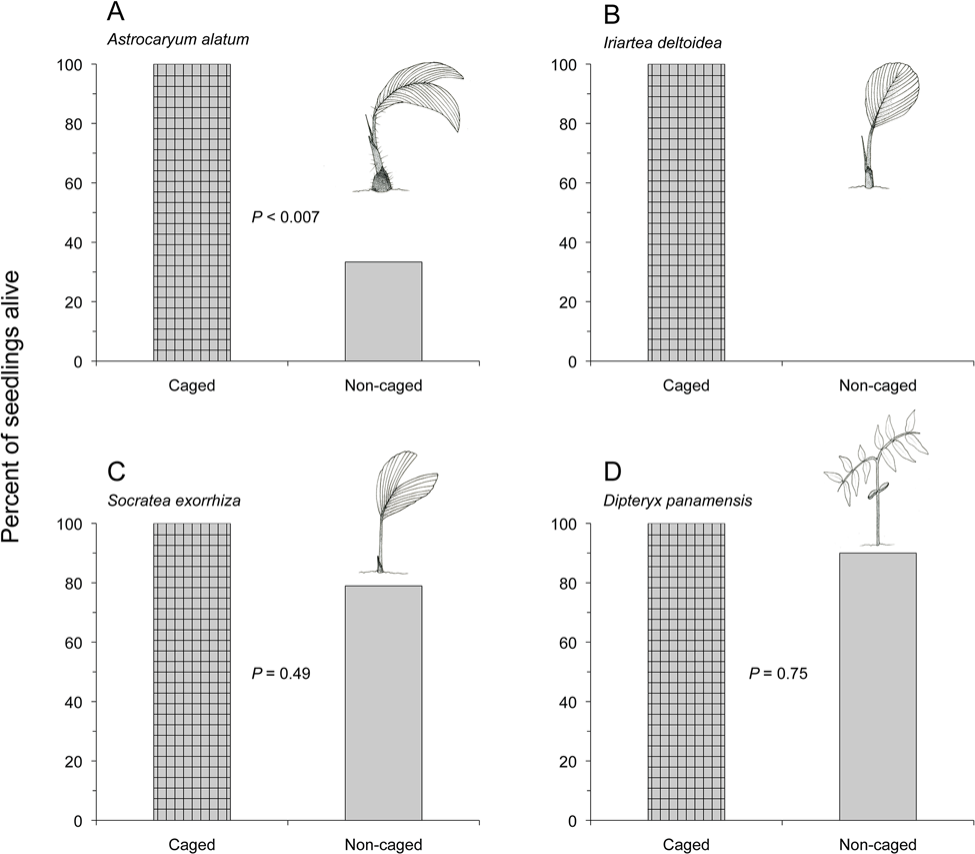
Percent of caged seedlings (protected from mammals) and non-caged seedlings (accessible to mammals) alive after 140 d in the forest. Sample sizes: *A*. *alatum* (A) *n* = 18 pairs, *I*. *deltoidea* (B) *n* = 17 pairs, *S*. *exorrhiza* (C) *n* = 19 pairs, and *D*. *panamensis* (D) *n* = 20 pairs.

Only two seedling species exposed to peccaries and agoutis were negatively affected by mammal granivory. One hundred percent of seedlings protected from mammals (caged treatment) survived through the 140 d study period. Seedlings of *A*. *alatum* and *I*. *deltoidea* that were exposed to mammals (uncaged treatment) suffered higher levels of mortality than caged seedlings (*A*. *alatum*: χ^2^ = 7.35, df = 1, *P* < 0.007; *I*. *deltoidea*: no statistical test needed for obvious difference between treatments; Fig 7). In contrast, very few non-caged seedlings of *S*. *exorrhiza* and *D*. *panamensis* were killed by mammal herbivores; the number of seedlings surviving at the end of the study did not significantly differ between caged and non-caged seedling treatments (*S*. *exorrhiza*: χ^2^ = 0.47, df = 1, *P* = 0.49; *D*. *panamensis*: χ^2^ = 0.11, df = 1, *P* = 0.75; Fig 7).

## Discussion

Using an experimental approach, I found that hoarding by agoutis was beneficial to most of the tree species used in this study, but negatively affected some aspects of plant survival. Seed caching protected seeds of all palm species (*A*. *alatum*, *I*. *deltoidea*, and *S*. *exorrhiza*) from detection and subsequent infestation by invertebrates. All observed invertebrate infestation was by scolytine beetles from the genus *Coccotrypes*. Females of *Coccotrypes* spp. lay 1–100 eggs within a single palm seed [7]. Seed death from this infestation likely depends on location and intensity of attack by *Coccotrypes*. Beetles that bore through the embryo kill the seed by preventing germination, whereas beetles that bore through endosperm only remove nutrient source and seed viability remains intact [35–37]. Also, infested seeds that contain high numbers of beetles are less likely to germinate and produce seedlings than seeds infested with low numbers of *Coccotrypes* individuals [7]. Palm seed infestation by *Coccotrypes* beetles occurs on the forest floor rather than in the canopy (post-dispersal seed predation). This is likely because of the inability of these small beetles to penetrate exocarp and pulp present on unripe fruits of *A*. *alatum*, *I*. *deltoidea*, and *S*. *exorrhiza* in the canopy [5]. Once these fruits mature, they shed their exocarp and pulp and seeds fall to the ground where they are exposed to *Coccotrypes* infestation [38]. Contrary to expectation, no infestation by bruchid beetles, a common invertebrate predator of large seeds in lowland neotropical forests [5, 6], was observed in seeds during this study. Loss of seed endosperm may not result in seed death (nonlethal predation), but seeds with large amounts of endosperm removed by invertebrates have lower germination success and smaller resultant seedlings than intact seeds [36, 39]. Insect attack also opens the seed coat to fungi and other pathogens and potentially causing dehydration [36]. Partial endosperm removal from seeds of *S*. *exorrhiza* by *Coccotrypes* resulted in stunted growth of seedlings because these insects removed the nutrient source available for seedling development.

Hoarding had no effect on levels of *D*. *panamensis* fruit infestation by invertebrate seed predators because no fruits of this species were infested, regardless of hoarding treatment. It is likely that *Coccotrypes* beetles were unable to penetrate the thick (5–7 mm), stony endocarp of *D*. *panamensis* fruit pods and gain access to the oily pulp and seed within. It is also possible that *Coccotrypes* beetles are not natural seed predators of *D*. *panamensis*. With the exception of *D*. *panamensis*, hoarding by agoutis has the potential to protect seeds from invertebrate attack, thereby allowing hoarded palm seeds to escape detection by insects, potentially germinate, and grow if they are not recovered by the hoarding animal.

Hoarding positively affected all seed species used in this study by effectively protecting buried diaspores from detection and removal by vertebrate seed predators. Agoutis consume and potentially disperse large seeds via hoarding, whereas peccaries consume and destroy most of the large seeds that they encounter on the forest floor [10, 11]. Although peccaries and other neotropical ungulates can disperse seeds that pass through their guts intact [10], in this study, seeds removed by peccaries were likely consumed and killed. Detailed information regarding the fates of *A*. *alatum*, *I*. *deltoidea*, *S*. *exorrhiza*, and *D*. *panamensis* seeds removed by peccaries and agoutis in a concurrent study is considered in [11].

Seeds not protected from invertebrates by hoarding had high levels of infestation and low germination success. No infested seeds of *A*. *alatum* or *I*. *deltoidea* successfully germinated and only 8% of *S*. *exorrhiza* seeds infested by *Coccotrypes* beetles germinated after 120 d. Hoarding improved palm seed germination success because hoarded seeds were not infested by *Coccotrypes* beetles. However, hoarding of *D*. *panamensis* fruits negatively affected germination success. Non-hoarded fruits exposed on the soil surface had high germination success (89%) while only 7% of hoarded seeds germinated. All non-germinating seeds had rotted within the fruit pod after 50 d of burial 5 cm below the soil. Previous studies have found that seed germination and seedling emergence are hindered if seedlings cannot penetrate the deep soil or leaf litter under which they are buried [40, 41]. It is likely that the mean depth of agouti hoards, as well as soil compaction, prevented *D*. *panamensis* fruit dehiscence and radicle and plumule emergence, trapping excess moisture within the unopened pod, and rotting the seed.

In this study, the survival of seedlings exposed to mammals differed among plant species. While peccaries and agoutis presumably killed non-caged seedlings of all four tree species, only seedlings of *A*. *alatum* and *I*. *deltoidea* had significantly different levels of mortality between caged (protected from mammals) and non-caged (exposed to mammals) treatments. Most non-caged seedlings died from uprooting and subsequent seed predation as opposed to trampling or herbivory by terrestrial mammals. Small seedlings may be acting as cues signaling underground food resources (seeds) for mammal granivores [12, 29, 30]. For some seedling species, removal of an attached seed may not result in seedling death [35], however uprooting and seed removal by peccaries and agoutis of seedlings of *A*. *alatum* and *I*. *deltoidea* resulted in death for 100% of the affected plants. In tropical rain forests, invertebrates play a major role in the herbivory and subsequent mortality of young seedlings (e.g., [26, 42, 43]). In the present study, seedlings within mammal exclosures were accessible to invertebrates and fungal pathogens. At the end of 140 d, 100% of all caged seedlings had survived and retained no evident meristem or leaf damage by invertebrate herbivores. Surprisingly, in this study, insects and fungal pathogens had negligible effects on the survival of young seedlings of *A*. *alatum*, *I*. *deltoidea*, *S*. *exorrhiza*, and *D*. *panamensis*. Perhaps young seedlings of these species possess tough leaves or secondary compounds that deter fungus and insect attack [42].

To prevent herbivory by mammals, some seedlings exhibit physical or chemical defenses [42, 44]. *Astrocaryum alatum* seedlings possess spines covering their leaves and stems. Although spines are generally thought to deter herbivory by mammals [44], the spines of *A*. *alatum* seedlings offered little defense against seed predation and subsequent seedling destruction by agoutis and peccaries (72% of seedlings exposed to mammals were killed within 140 d). Terrestrial mammal granivores were able to circumvent these physical defenses, uproot young plants, remove attached seeds, and incidentally kill exposed seedlings.

After 140 d, all seedlings of *I*. *deltoidea* exposed to mammals were killed. Seedlings of this tree species have no spines or apparent physical defenses against herbivory aside from thick leaves that may decrease palatability [42, 44]. It is interesting to note that in this study, all palm seedling deaths occurred due to predation of attached seeds and resultant seedling uprooting rather than by direct herbivory (i.e., consumption of meristem or leaf area). Young leaves of these seedlings may possess physical or chemical anti-herbivory defenses. In contrast, seedlings of *D*. *panamensis* killed by mammals (*n* = 2) died from leaf and cotyledon consumption. Only 10% of *D*. *panamensis* seedlings available to mammals were consumed and killed within the census period. The low mortality of *D*. *panamensis* seedlings observed in this study contrasts with very high mortalities found in previous research (81% mortality in 12 mo, [27]; 67%–88% in 13 mo, [45]). The low amount of vertebrate herbivory on young *D*. *panamensis* seedlings observed in this study may be attributed to shorter exposure times (5.5 mo) or low densities of conspecific seedlings near the experimental pairs. Seedling pairs were separated by at least 300 m and were not necessarily located near naturally occurring conspecifics. Previous studies have found that seedlings of this species experience high density-dependent mortality [26, 45, 46] and it is likely that the seedling densities of *D*. *panamensis* created by this study were too low to elicit a strong herbivore response.

This study reinforces the idea that the seed and seedling stages of plants are highly susceptible to mortality from seed-eating insects and mammals. Seeds not protected from invertebrate and vertebrate seed predators suffered very high mortality rates. Scatter hoarding by agoutis positively affected palm seeds by protecting them from both insect and mammal predation, thereby improving subsequent seed germination and seedling growth. However, for *D*. *panamensis*, there is a trade-off in survival: hoarding protects fruits from vertebrate predation (positive effect) but prevents seed germination (negative effect). Although *D*. *panamensis* fruits hoarded by agoutis have a higher likelihood of escaping mammal seed predators than non-hoarded fruits, buried seeds are unlikely to germinate and produce seedlings that can emerge from agouti caches. This unexpected outcome suggests that hoarding cannot be assumed to always benefit seeds, as numerous past studies have asserted, and that other animal species may play more important roles in the dispersal of this species. In conclusion, scatter hoarding can be beneficial or detrimental to plants by having a significant effect on rates of seed infestation by insects and predation by mammals, germination success, and seedling growth. Agouti hoarding behavior has the potential to strongly influence seed and seedling survival, recruitment, and potential tree establishment in forests where these mammals are common.
